# Multi-Scale Attention Networks with Feature Refinement for Medical Item Classification in Intelligent Healthcare Systems

**DOI:** 10.3390/s25175305

**Published:** 2025-08-26

**Authors:** Waqar Riaz, Asif Ullah, Jiancheng (Charles) Ji

**Affiliations:** 1 Institute of Intelligent Manufacturing Technology, Shenzhen Polytechnic University, 4089 Shahe West Road, Shenzhen 518055, China; riazwaqar@szpu.edu.cn (W.R.); asifkh@szpu.edu.cn (A.U.); 2 Shenzhen Institutes of Advanced Technology, Chinese Academy of Sciences, Shenzhen 518055, China

**Keywords:** clinical inventory classification, image sensing in healthcare, AI-enabled healthcare, mask RCNN, YOLO, EfficientDet-BiFormer integration

## Abstract

The increasing adoption of artificial intelligence (AI) in intelligent healthcare systems has elevated the demand for robust medical imaging and vision-based inventory solutions. For an intelligent healthcare inventory system, accurate recognition and classification of medical items, including medicines and emergency supplies, are crucial for ensuring inventory integrity and timely access to life-saving resources. This study presents a hybrid deep learning framework, EfficientDet-BiFormer-ResNet, that integrates three specialized components: EfficientDet’s Bidirectional Feature Pyramid Network (BiFPN) for scalable multi-scale object detection, BiFormer’s bi-level routing attention for context-aware spatial refinement, and ResNet-18 enhanced with triplet loss and Online Hard Negative Mining (OHNM) for fine-grained classification. The model was trained and validated on a custom healthcare inventory dataset comprising over 5000 images collected under diverse lighting, occlusion, and arrangement conditions. Quantitative evaluations demonstrated that the proposed system achieved a mean average precision (*mAP*@0.5:0.95) of 83.2% and a top-1 classification accuracy of 94.7%, outperforming conventional models such as YOLO, SSD, and Mask R-CNN. The framework excelled in recognizing visually similar, occluded, and small-scale medical items. This work advances real-time medical item detection in healthcare by providing an AI-enabled, clinically relevant vision system for medical inventory management.

## 1. Introduction

The accelerating demand for artificial intelligence (AI) in smart healthcare has driven the adoption of intelligent robotic systems that assist with essential operational tasks, particularly through advanced vision-based technologies. AI-driven robotics now play a crucial role in supporting complex healthcare logistics, including automated inventory inspection and stock-level monitoring through real-time image analysis [1]. In such environments, accurate recognition and classification of medical items, including pharmaceuticals and emergency supplies, are indispensable for ensuring operational continuity and reducing manual handling errors. AI applications have been widely adopted in vision-related analysis due to their capability in segmentation, classification, and visual interpretation tasks [2,3]. However, within the context of healthcare inventory management, one of the persisting challenges lies in reliably identifying and retrieving items across diverse storage conditions, such as pharmacy cabinet units [3]. While many existing systems focus on diagnostic imaging or high-level environmental understanding [4], fewer tools are designed for shelf-level inventory item recognition under cluttered conditions.

Manual inventory processes, often slow and error-prone, are inadequate for the time-sensitive nature of healthcare delivery. Misplaced or misclassified medical supplies can delay treatment and lead to severe patient outcomes. Deep learning-based object detection frameworks, such as YOLO, Faster R-CNN, SSD, and U-Net, have revolutionized general image detection tasks [5,6,7,8,9]. Yet, these models show limitations in small-object detection, handling occlusion, and achieving fine-grained classification under real-world healthcare conditions. To address these gaps, we propose a hybrid deep learning architecture that unites EfficientDet with its BiFPN for scalable multi-scale feature aggregation, BiFormer with bi-level routing attention for enhancing contextual awareness, and ResNet-18 for robust fine-grained recognition [10,11,12]. 

**Figure 1:**
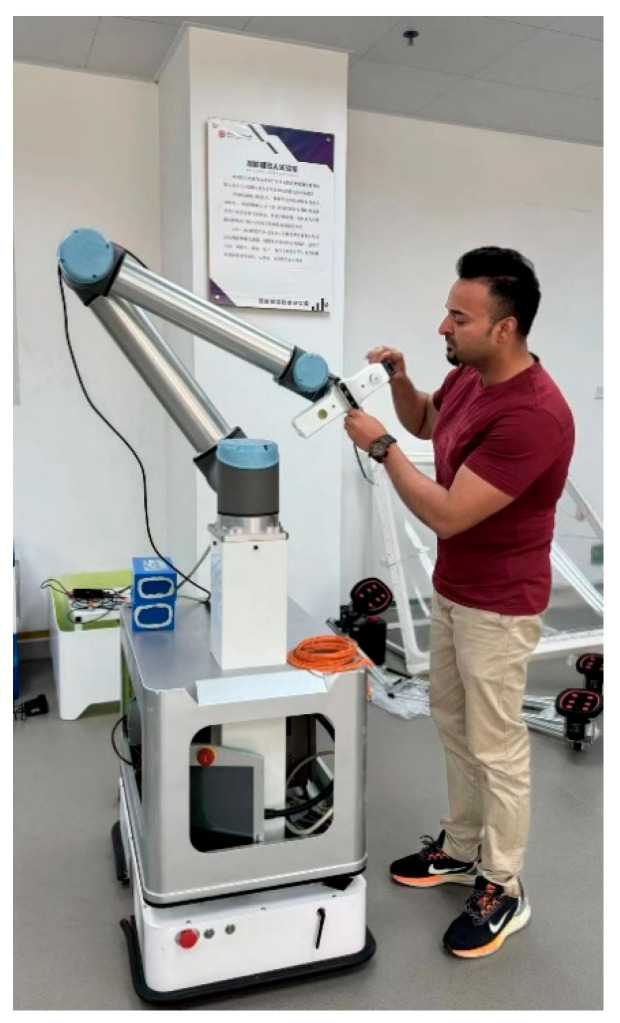
Hardware structure of the mobile robot designed to deploy in the proposed system [17].

This study is part of a broader AI-enabled intelligent healthcare system for mobile robot as shown in Figure 1, focusing on the accurate detection of medical items through deep learning-driven vision for automated recognition and tracking in clinical inventory management. Unlike conventional detectors, this architecture leverages Transformer-based attention refinement via BiFormer, triplet loss embeddings, and Online Hard Negative Mining (OHNM) to improve the classification of visually similar medical items and reduce false positives in cluttered settings [10,11,12,13,14]. The integration of attention-based feature refinement and metric learning substantially reduces classification errors and enhances retrieval accuracy in cluttered medical environments. Despite the advancements, most current systems focus on clinical diagnostics or broad scene understanding, leaving a clear gap in tools that support precise, real-time inventory recognition in healthcare settings. Our work addresses this gap by developing a purpose-built architecture optimized for shelf-level item detection and categorization. This system is designed not to impact clinical outcomes but to enhance intelligent inventory management in healthcare logistics, reduce human error in inventory tracking, and support the automation of routine management tasks. By doing so, it contributes directly to the broader goal of AI-enabled intelligent healthcare operations. Evaluated on a carefully curated dataset that simulates real healthcare shelf conditions, including occlusion scenes and heterogeneous lighting, the proposed model shows marked improvements over existing detection and classification baselines [14,15,16]. The contributions of this work are as follows:

- We propose a novel hybrid deep learning pipeline that combines EfficientDet’s BiFPN, BiFormer’s attention refinement, and ResNet-18 with triplet loss for robust medical item recognition.
- We introduce a custom dataset of 5000+ annotated medical inventory images collected under real-world healthcare storage conditions, including occlusion, diverse lighting, and random clutter.
- We demonstrate the model’s real-time inference capability on embedded healthcare robots, achieving 94.7% top-1 accuracy and 83.2% *mAP*@0.5:0.95, with rigorous ablation and uncertainty analysis.
- We incorporate online hard negative mining and class-balanced focal loss to address fine-grained visual similarity and dataset imbalance in a healthcare inventory setting.

The remainder of this paper is structured as follows: the Section 2 reviews deep learning methods in medical imaging and healthcare robotics, as well as image classification for inventory; the Section 3 describes our EfficientDet-BiFormer-ResNet framework; the Section 4 details our performance evaluation; and the Section 5 summarizes conclusion and future directions.

## 2. Related Work

The increasing reliance on automation and AI-driven robotics in healthcare has driven extensive research into computer vision-based object detection for inventory management and autonomous robotic systems. Traditional object detection methods, including HOG (Histogram of Oriented Gradients) and SIFT (Scale-Invariant Feature Transform), were widely used before deep learning advancements. However, these techniques failed to generalize well to complex medical environments due to their handcrafted feature extraction limitations [18]. With the emergence of deep learning-based object detection models, such as YOLO, Faster R-CNN, and SSD, object detection has seen significant improvements in accuracy, speed, and adaptability. However, these models still face challenges in cluttered and occluded shelf conditions where objects exhibit high visual similarity [19]. Later YOLO models demonstrate high-speed real-time processing capabilities but struggle with small-object detection due to their reliance on single-shot detection pipelines [20]. Faster R-CNN, while achieving higher accuracy through region-based proposals, suffers from computational inefficiency, making it unsuitable for real-time applications [21]. SSD provides a trade-off between speed and accuracy, but its performance deteriorates in high-occlusion settings, making it inadequate for cluttered objects placed on shelves [22].

Over the past years, several researchers have focused on an optimized detection framework, named EfficientDet, which integrates BiFPN to enhance multi-scale feature aggregation [23]. EfficientDet was successfully deployed in small object detection applications, demonstrating superior performance in detecting small and occluded objects, which is crucial for identifying medicine boxes and medical supplies in healthcare environments [24]. One of the fundamental challenges in automated inventory recognition is differentiating between visually similar items. CNN-based object detectors primarily rely on spatial feature maps, which can lead to feature redundancy and misclassification when handling high-similarity objects [25]. To address this, recent research has explored Transformer-based architectures, such as Vision Transformers (ViTs) and BiFormer, which introduce self-attention mechanisms for improved feature representation [26]. ViTs demonstrate significant improvements in long-range dependency modeling but often require extensive computational resources, limiting their feasibility for real-time applications [27]. But BiFormer, a more lightweight Transformer variant, integrates bi-level routing attention, allowing selective feature refinement and improved contextual understanding in dense environments. When combined with EfficientDet’s detection backbone, BiFormer enhances feature refinement and reduces false positives, enabling better object differentiation even in complex shelf conditions [28,29].

Beyond detection, our proposed method requires robust classification models capable of recognizing medicine and medical supplies in shelves. CNN-based classification architectures, including VGG, Inception, and ResNet, have been widely utilized for medical image recognition [30]. Among these, ResNet-18 is lightweight and has been particularly effective due to its residual learning mechanism, which mitigates the vanishing gradient problem and enables efficient deep network training using approaches such as triplet loss-based feature embeddings [31,32]. Triplet loss improves model robustness by learning a discriminative feature space, ensuring that object classification is effectively separated in feature space. Additionally, several existing object detection models lack the computational efficiency required for real-time applications, limiting their applicability in mobile robot applications [33,34].

Current CNN-based classification systems struggle with highly similar objects, leading to misclassification issues in real-world environments [35]. Huo et al. presented a Res2Net Transformer hybrid for high-resolution remote sensing scene classification, which leverages multi-scale local features and global attention for fine-grained categorization. Its handling of spatially distributed, visually similar classes parallels our cluttered healthcare inventory setting [36]. Hybrid designs typically extract local textures with CNN stages and model global relations with Transformer blocks, which helps under visual ambiguity (e.g., low-contrast defects; night/rain driving). In industrial inspection, Swin/Transformer hybrids with conv pyramids have shown superior defect localization compared to pure CNNs by capturing small repetitive patterns and their global layout; one example is variant Swin for surface defects and Defect–Transformer hybrids [37]. Inspired by these studies, we present an EfficientDet-BiFormer-ResNet framework optimized for these healthcare robotic vision systems. This hybrid approach ensures accurate and efficient object detection using EfficientDet’s BiFPN-based feature aggregation unified with BiFormer’s context-aware feature refinement, reducing misclassification of visually similar objects. We additionally incorporate robust classification using Res-Net-18 with triplet loss and OHNM, which improved fine-grained recognition in our medicine and medical supplies scenario, enabling real-time decision-making for medical inventory tracking and retrieval. The framework holds significant promise for deployment in healthcare robots and automated pharmacy systems, contributing directly to safer, faster, and smarter clinical decision-making.

## 3. Methodology

### 3.1. Overview

**Figure 2:**
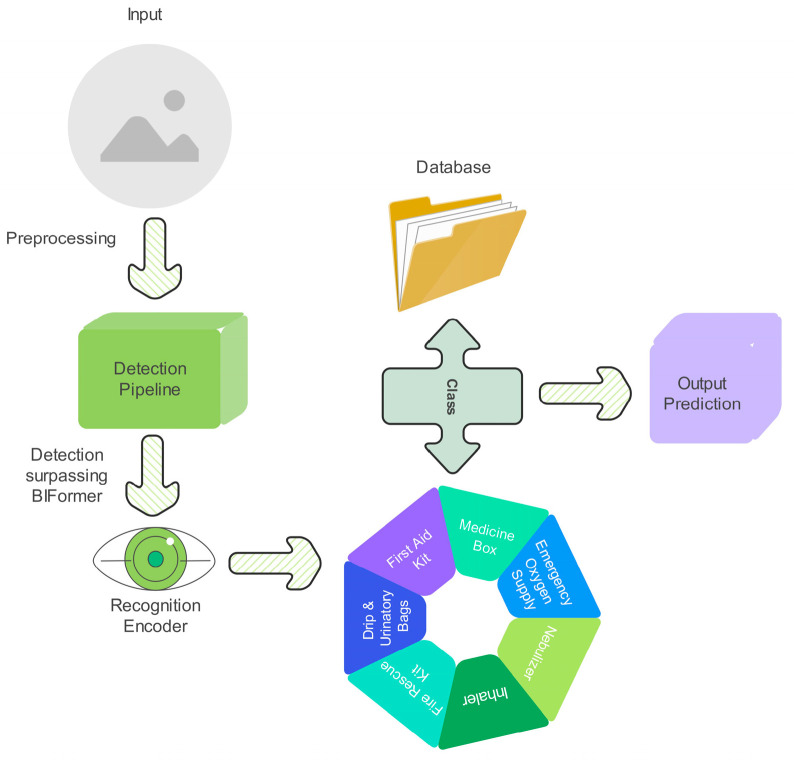
Block diagram of the proposed framework, which is further elaborated in subsequent figures.

The proposed model is designed for an autonomous healthcare mobile robot that detects and recognizes medicines and medical supplies on healthcare shelf settings using a two-stage deep learning framework, as shown in Figure 2. The pipeline processes input image frames in real time, starting with preprocessing, followed by object detection using EfficientDet, feature refinement via BiFormer, and fine-grained classification using ResNet-18 with triplet loss and OHNM. The goal is to accurately localize, distinguish, and recognize medical supplies and medicines while operating in cluttered environments where objects exhibit occlusions, visual similarity, and orientation variations. The system begins by capturing high-resolution image frames from the robot’s onboard camera, which are resized, normalized, and preprocessed before being passed into the EfficientDet-based object detection module. Given an input image X, EfficientDet extracts multi-scale feature representations using its Bidirectional Feature Pyramid Network (BiFPN) and compound scaling approach, ensuring improved localization precision for delicate and occluded objects. The model outputs bounding box predictions $B={\left\{\left(x_{i},y_{i},w_{i},h_{i},c_{i}\right)\right\}}_{I=1}^{N}$, where $\left(x_{i},y_{i}\right)$ are the bounding box coordinates, $\left(w_{i},h_{i}\right)$ are the bounding box width and height, and $\left(c_{i}\right)$ represents the class confidence score. Unlike conventional CNN-based detectors, which process fixed feature maps, BiFPN enables bidirectional feature fusion, computed as:

(1) $$F_{l}=\sum_{i=1}^{n}w_{i}.U(F_{i})$$

where $F_{l}$ and $F_{i}$ are feature maps from different network stages, $w_{i}$ are weight matrices, allowing the network to adaptively refine feature importance, and $U(F_{i})$ represents upsampled feature maps from adjacent. Once objects are detected, their region proposals are passed to the BiFormer-based feature refinement module. Standard CNNs suffer from feature redundancy and spatial misalignment, especially in healthcare shelf environments where objects have minor differences in labels. BiFormer improves classification by applying bi-level routing attention, selectively attending to discriminative spatial features. Given input features *X*, the attention mechanism is computed, which filters irrelevant features and enhances classification robustness in cluttered scenes.

Finally, the refined feature maps are fed into the ResNet-18-based classification module, which learns a discriminative embedding space for fine-grained recognition. Instead of using cross-entropy loss, the system leverages triplet loss for metric learning, ensuring medicines with slightly similar packaging but different shapes or sizes remain separable in feature space. The triplet loss function is formulated as:

(2) $$L_{triplet}=\frac{1}{N}\sum_{i=1}^{N}\underset{i}{\max}\left(0,d\left(x_{a},x_{p}\right)-d\left(x_{a},x_{n}\right)+\alpha \right)$$

where N is the no. of training samples; α denotes the fixed margin parameter, a positive constant that enforces a minimum separation between the anchor positive and anchor negative distances in the embedding space, thereby encouraging discriminative feature separation; $x_{a}$, $x_{n}$, and $x_{p}$ are the anchor sample (query medicine image), positive sample (same category as anchor), and negative sample (different category), respectively; $d\left(x_{a},x_{p}\right)$ and $d\left(x_{a},x_{n}\right)$ are the Euclidean distance function, ensuring that similar samples remain close while dissimilar samples are separated; and α is a margin parameter to enforce feature separation. Additionally, OHNM is used to prioritize difficult negative samples, further refining classification accuracy. The final recognized objects are mapped back to their bounding boxes and integrated into the robot’s inventory management system, updating healthcare environment databases for automated medicine tracking and retrieval. The model achieves over 20 FPS on a Jetson AGX Orin, enabling real-time deployment on the healthcare mobile robot. This deep learning-driven automation system enhances efficiency and reduces human dependency.

### 3.2. Dataset Collection and Preprocessing

**Figure 3:**
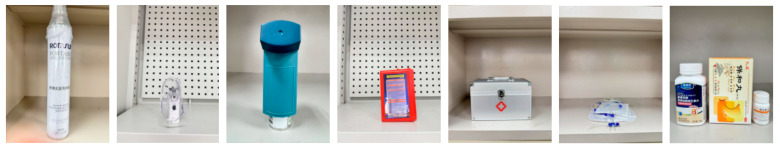
The collection of medicine and medical supplies for each class category, including emergency oxygen supplies, nebulizers, inhalers, fire rescue kits, first-aid kits, grip and urinatory bags, and medicine boxes (left to right).

**Figure 4:**
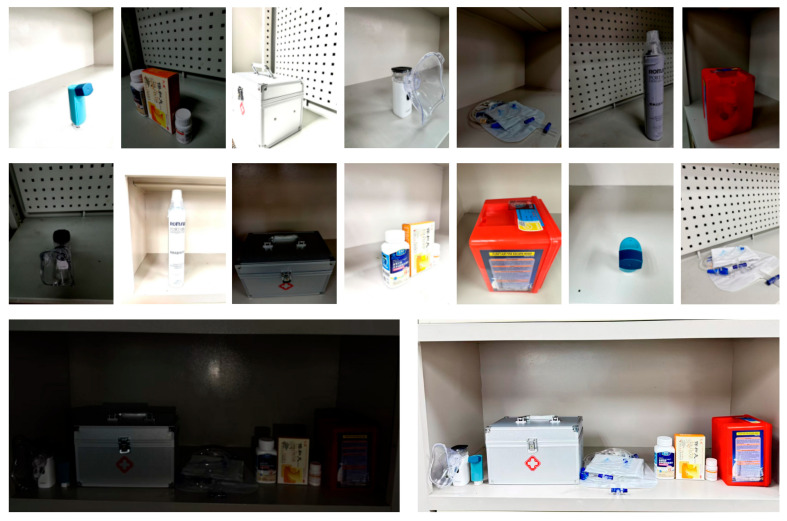
Capturing types with different angles and lighting conditions.

To ensure the robustness and generalization of proposed healthcare mobile robot’s vision-based detection and recognition system, a custom dataset was meticulously curated, comprising high-resolution images of medicine boxes and medical supplies, including medicine boxes, first aid kits, emergency oxygen supplies, inhalers, nebulizers, fire rescue kit, and drip and urinatory bags, captured under diverse real-world conditions, as shown in Figure 3 and Figure 4.

Unlike publicly available datasets, which often lack occlusion-heavy, cluttered, and dynamically arranged shelf images found in real healthcare environments, this dataset was self-collected to simulate healthcare unit storage conditions. The dataset was compiled using a multi-camera setup, ensuring diverse perspectives and adaptability for real-time robotic vision. The image acquisition was conducted using a healthcare shelf type in various settings, capturing, after all data augmentation techniques, a total of 5000+ images of different medicines and medical supplies arranged in different lighting conditions (natural daylight, artificial healthcare lighting, low-light emergency room environments), varying shelf arrangements (stacked medicines, randomly placed medical supplies, rotated boxes), and occlusion and overlapping cases, simulating real-world cluttered healthcare settings. Each medicine or medical supply was captured from multiple angles to ensure robust feature learning, allowing the system to recognize objects even when partially visible. Table 1 illustrates the details about the metadata (such as resolutions, per-class counts, camera specs).

The dataset was manually annotated using bounding boxes and class labels, ensuring precise ground truth mapping for EfficientDet-based object detection and ResNet-based fine-grained recognition. Formally, the dataset was defined as a set of labeled image samples:

(3) $$D={\left\{\left(x_{i},y_{i}\right)\right\}}_{i=1}^{N}$$

where $x_{i}\in R^{H\times W\times C}$ represents an image sample of height *H*, width *W*, and channels *C*, and $y_{i}$ denotes the corresponding object category label for supervised training. Before training, all collected images underwent a standardized preprocessing pipeline to ensure uniformity, noise reduction, and optimal feature extraction across different lighting and occlusion conditions. The preprocessing steps include the following:

1. Image Resizing and Normalization: All images are resized to a fixed dimension of 1024 × 1024 pixels to match the input requirements of EfficientDet and ResNet-18. Pixel values are normalized to a [0, 1] range using min-max normalization, ensuring stable gradient updates during training:

(4) $$X_{norm}=\frac{X-X_{min}}{X_{max}-X_{min}}$$

2. Data Augmentation and Illumination: Images are randomly rotated within ${\pm 30}^{\circ }$ flipped (horizontal and vertical) and cropped to simulate variations in real-world medicine shelf orientations. Brightness and contrast adjustments are applied, where U(a,b) denotes a uniform distribution and a and b in U(a,b) are the lower and upper bounds, ensuring adaptation to different healthcare environment lighting scenarios:

(5) $$X_{new}=X\times \alpha +\beta ,\alpha \sim U\left(0.8,1.2\right),\beta \sim U(-20,20)$$

1. Noise Injection: Gaussian noise with variance $\sigma^{2}=0.02$ is applied to simulate real-world motion blur and sensor noise in robotic navigation.
2. Bounding Box Refinement for EfficientDet: To optimize object detection, bounding boxes are refined using *IoU*-based (Intersection over Union) filtering, discarding low-confidence annotations and ensuring accurate region proposals:

(6) $$IoU=\frac{B_{1}\cap B_{1}}{B_{1}\cap B_{2}}$$

where $B_{1}$ and $B_{2}$ are overlapping bounding boxes.

Hard Sample Mining for Recognition Training: Difficult-to-classify images (e.g., medicine and medical supplies that may vary in size but with minor visual differences) are prioritized in mini-batches, ensuring ResNet-18 learns fine-grained details more effectively.

Additionally, to prevent overfitting and ensure unbiased evaluation, the dataset is split into three subsets, including the training set (70%), used for model learning, the validation set (20%), used to fine-tune hyperparameters, and the test set (10%), used for performance evaluation under unseen conditions. This structured data collection and preprocessing pipeline ensures that the EfficientDet-BiFormer-ResNet hybrid model achieves high accuracy, robust generalization, and real-time inference capabilities for healthcare robotic applications.

### 3.3. Object Detection Module

Traditional object detectors, such as Faster R-CNN, SSD, and YOLO variants, have demonstrated significant success in general object detection tasks, yet they struggle in specialized domains. YOLO-based models, despite their high-speed inference, often compromise on detection accuracy for small and overlapping objects. SSD, on the other hand, lacks the hierarchical feature fusion capabilities required for handling scale variations, a critical factor in detecting small medicine boxes placed alongside larger medical supplies or other supplies. The object detection module in our proposed model serves as the first stage in the proposed vision pipeline, responsible for the precise localization and detection of medicine and medical supplies within complex healthcare shelf environments. Given the cluttered arrangement, variable lighting, and frequent occlusions in medical storage settings, an optimized detection framework is required to ensure robust multi-scale detection with real-time efficiency. To achieve this, EfficientDet is employed as the backbone detection model, leveraging Bidirectional Feature Pyramid Networks (BiFPNs) for multi-scale feature aggregation alongside Biand compound scaling for optimal trade-offs between accuracy, computational efficiency, and model depth.

**Figure 5:**
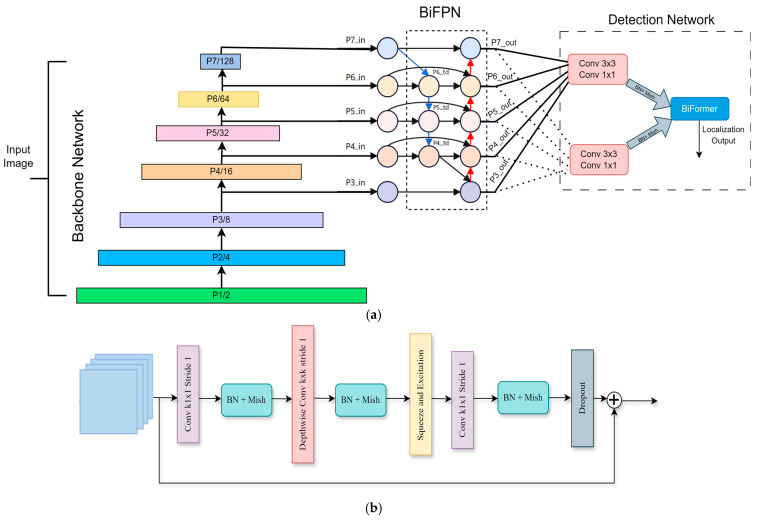
(**a**) Illustrates the detection architecture, including the backbone and object localization and detection. (**b**) MBconv block representation.

EfficientDet addresses these challenges through two primary architectural innovations in our model, including BiFPN, which is a multi-scale feature aggregation mechanism that enhances small-object detection and improves spatial feature propagation across different levels of the feature hierarchy. The EfficientDet architecture uniquely integrates bidirectional and cross-scale connectivity by creating supplementary connections between original input and output nodes at corresponding hierarchical levels. This approach involves stacking identical feature layers multiple times, effectively balancing detection precision with computational efficiency. As clearly demonstrated in the EfficientDet algorithm depicted in the accompanying network diagram shown in Figure 5a, EfficientDet is structured into three distinct stages. The first is feature extraction as the backbone, which extracts critical features by leveraging Neural Architecture Search (NAS) in combination with compound scaling strategies. The next stage is Feature Integration, where BiFPN combines cross-layer features, significantly enhancing detection performance through enriched multi-scale representations. The last stage, Detection and Localization, is performed by dedicated classification and regression networks, which accurately predict the object class and pinpoint object locations.

Furthermore, the foundational and terminal layers within EfficientDet typically consist of simple convolutional layers combined with batch normalization and the Mish activation function, primarily using convolutional kernels sized 3 × 3 and 1 × 1. In the intermediate layers, the architecture consistently employs a series of convolutional modules known as Mobile Inverted Bottleneck Convolution (MBConv) blocks. Initially, each MBConv block expands the dimensions of the input features through a convolutional operation utilizing 1 × 1 kernels. Subsequently, the expanded features undergo depthwise separable convolutions (DSCs) performed with either 3 × 3 or 5 × 5 kernels. The block then concludes with the integration of a Squeeze-and-Excitation Network (SENet), which compresses the dimensionality back down via an additional 1 × 1 convolution, thereby refining the feature representation effectively. Figure 5b provides a visual representation of this detailed structure. Additionally, EfficientDet utilizes compound scaling methods to fine-tune network dimensions, including depth, width, and image resolution to achieve optimal efficiency. These methods yield a compact yet powerful architecture suitable for deployment in environments with limited computational resources, such as robotic platforms. The core part of EfficientDet is BiFPN, which integrates bidirectional pathways with advanced scaling mechanisms. This core innovation not only enhances the model’s learning efficiency during training but also ensures robust real-time processing and highly accurate object detection. BiFPN introduces bidirectional cross-scale connections, ensuring that features from multiple resolutions interact effectively, enhancing detection accuracy for small objects in the healthcare shelf setting. Formally, for each feature level l, the BiFPN representation is computed as Equation (1) $F_{l}=\sum_{i=1}^{n}w_{i}.U(F_{i})$,

This mechanism allows the detector to dynamically prioritize critical features in cluttered shelves, preventing small objects, such as individual medicine boxes, from being ignored in dominant background elements.

#### 3.3.1. Bounding Box Regression and Training Optimization

EfficientDet uses an anchor-free object detection strategy, where bounding boxes are generated based on scale-invariant keypoints instead of predefined anchor sizes. This reduces computational redundancy while ensuring that detection generalizes well to varying shelf layouts. The detection module predicts a set of bounding boxes *B* and confidence scores *c*, defined as:

(7) $$B={\left\{\left(x_{i},y_{i},w_{i},h_{i},c_{i}\right)\right\}}_{i=1}^{N}$$

where *N* donates as the number of instances, $x_{i}\;and\;y_{i}$ are the center coordinates of the detected object, $w_{i}$ denotes the width and height of the bounding box, and $c_{i}$ is the classification confidence score, indicating the probability of the object belonging to a particular class. The bounding box loss function $L_{bb}$ is defined below, where $B_{i}$ is the predicted bounding box and ${\hat{B}}_{i}$ is the ground truth annotation:

(8) $$L_{bb}=\sum_{i=1}^{N}SmoothL1{(B}_{i}-{\hat{B}}_{i})$$

The EfficientDet model was trained using a weighted focal loss function, which adjusts the loss contribution of hard and easy examples, ensuring that the detector focuses on difficult-to-detect shelf objects rather than being dominated by easily recognizable objects. This focal loss function is defined as:

(9) $$L_{focal}=\alpha {\left(1-p_{t}\right)}^{\gamma }log(p_{t})$$

where $p_{t}$ is the predicted probability for the target class, γ is the focusing parameter, controlling the importance given to misclassified samples. and α is a class-specific weighting factor, preventing dominance by majority classes. For real-time deployment in the healthcare mobile robot, EfficientDet was trained with the following hyperparameters: the Image Input Size was 1024 × 1024 and the batch size was 16. The Adam optimizer was used with cosine learning rate decay, with an Initial Learning Rate of 0.0002 and a total number of total training epochs of 100, which is discussed in detail in subsequent sections.

#### 3.3.2. BiFormer for Feature Refinement Module

Widely recognized for modeling long-range dependencies, the self-attention mechanism has become a key component in modern object detection frameworks [38]. However, its benefits come at the cost of a substantial memory footprint and considerable computational overhead. To mitigate these issues, researchers have introduced a variety of handcrafted sparse attention patterns [39] aimed at reducing model complexity. Although these patterns ease the computational burden, they still fail to fully capture long-range relationships. BiFormer contributes novelty by using dynamic sparse attention to selectively focus on discriminative details of each detected region. BiFormer differs significantly from traditional feature fusion layers like FPN or BiFPN, which apply static feature merging across resolutions. In contrast, BiFormer introduces bi-level routing attention, where each token first performs region-to-region selection followed by intra-region token-level refinement. This enables adaptive attention computation, which has been shown to outperform uniform attention mechanisms in visually cluttered scenarios. Zhu et al. demonstrated that BiFormer improves object differentiation in complex visual contexts by suppressing irrelevant spatial noise and enhancing semantically coherent regions, even in non-contiguous spatial layouts [11]. Such characteristics are particularly suited to our healthcare inventory scenes, where visual confusion (due to occlusion or similar packaging) can significantly hinder CNN-based methods. In our pipeline, BiFormer is positioned after EfficientDet’s BiFPN output, where it enhances contextual representation by selecting discriminative attention patterns aligned with visual semantics. Thus, even without maps, existing evidence supports its effectiveness as a mid-level context enhancer for fine-grained medical inventory differentiation. By routing attention to fine details, BiFormer helps the system confidently distinguish look-alike items, significantly reducing misclassification. This novel integration thus provides improved contextual feature refinement over EfficientDet alone, as evidenced by fewer false positives and higher *mAP* when the BiFormer is included (*mAP* increases from 71.4% to 83.2% in our ablation).

Although the EfficientDet-based detection module effectively localizes healthcare shelf objects, traditional CNN-based feature extractors often struggle to differentiate between visually similar medical items, especially when packaging variations are minimal. Many medicines exhibit identical box designs, minor color differences, or nearly indistinguishable text-based labels, making conventional recognition models prone to misclassification and false positives. To address this challenge, we incorporate BiFormer (Bi-level Routing Transformer) as a feature refinement module, which introduces context-aware attention mechanisms to enhance the discriminative capability of the vision pipeline.

**Figure 6:**
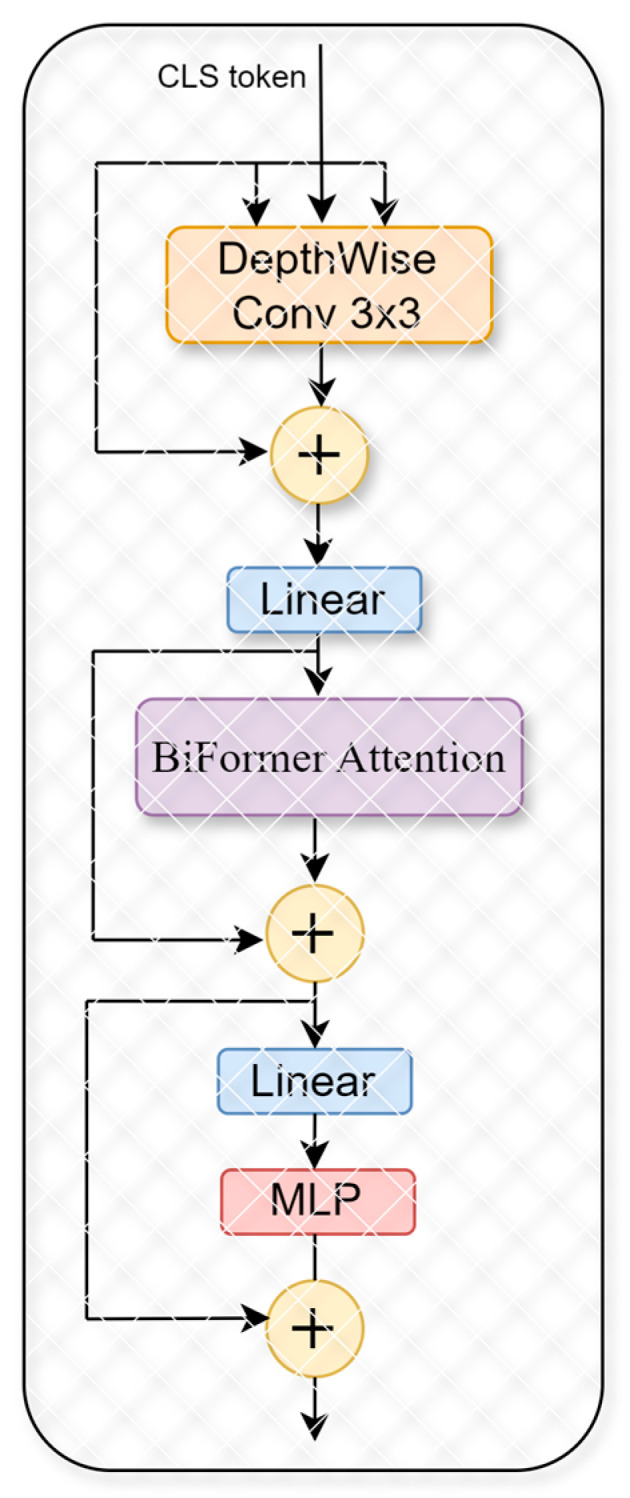
Overall attention mechanism with a bi-level routing block in BiFormer.

In this study, we utilize the improved generalized visual network framework of BifFormer. The fundamental innovation behind BiFormer lies in its two-stage attention mechanism inspired by [40]. Initially, it conducts a preliminary filtering of less relevant key-value pairs at a broader, regional scope; subsequently, it refines this attention at a more detailed, token-level granularity. This dual-stage approach not only allows the network greater adaptability in handling diverse content but also significantly boosts computational efficiency and minimizes memory requirements. Thus, the BiFormer model effectively preserves the strengths inherent in the Transformer architecture while also offering enhanced flexibility in content perception and resource management, as illustrated in Figure 6, which depicts the structure of the BiFormer model. BiFormer operates on a bi-level routing attention mechanism, ensuring that the model selectively focuses on the most informative spatial features.

Given an input feature map $X\in R^{H\times W\times C}$ derived from the EfficientDet backbone, BiFormer utilizes an attention-based feature selection technique. The approach operates by segmenting the feature map into *S* × *S* distinct regions without overlap. Each of these regions consists of multiple feature vectors, each characterized by a dimensionality denoted as $\frac{H\times W}{S^{2}}$. After reshaping the input map, the regions are partitioned into $X^{r}\in R^{S^{2}\times \;\frac{H\times W}{S^{2}}\times C}$, followed by a linear transformation to obtain $Q=X^{r}W^{q}$, $K=X^{r}W^{k},$ and $V=X^{r}W^{v}$, where $W^{q},\;W^{k},\;{andW}^{v}$ are the projection weights. A region-to-region affinity matrix is then computed by performing matrix multiplication on $Q^{r}K^{r}\in R^{S^{2}\times C}$, with each element of the resulting matrix, $A^{r}$, capturing the semantic relationship between regions:

(10) $$A^{r}=Q^{r}{\left(K^{r}\right)}^{T}$$

Unlike self-attention in conventional Transformers, which computes relationships between all tokens in an image, here, the bi-level routing strategy incorporates, in which tokens are first filtered based on priorities before attention is applied. This approach enables the network to selectively concentrate on highly informative areas, thus considerably lowering computational demands and simultaneously improving the accuracy of recognition tasks, particularly in densely populated medical shelves. The token-to-token attention mechanism operates by employing a region-specific routing strategy, where a routing index matrix, $I^{r}$, derived by selecting the top *k* indices from attention scores $\left(A^{r}\right)$, guides the formation of detailed attention interactions among tokens within these prioritized regions. These regions, though effectively targeted, might be dispersed widely across the entire spatial feature maps. The key $K^{g},\;V^{g}$ tensors are gathered by:

(11) $$K^{g}=gathered\left(K,I^{r}\right)$$

(12) $$V^{g}=gathered\left(V,I^{r}\right)$$

Subsequently, the gathered key-value pairs undergo a dedicated attention computation, resulting in the final output representation *Z*.

(13) $$Z=Att(Q,K^{g},V^{g}+LCE(V)$$

where *LCE*(*V*) denotes a local context enhancement component specifically designed to enrich the descriptive power of local context features, carefully balancing improved representation with computational efficiency.

### 3.4. Recognition Module

The final stage of the vision system pipeline is the recognition module, which is responsible for fine-grained classification of detected medicines and medical supplies. The ResNet-18 backbone was selected not only for its low inference latency on embedded robotic platforms, but also due to its proven balance of accuracy and computational efficiency in data-limited, fine-grained classification settings [41]. By coupling ResNet-18 with triplet loss and Online Hard Negative Mining, we achieve competitive discriminative performance without incurring the complexity and higher power demands of deeper architectures [42].

**Figure 7:**
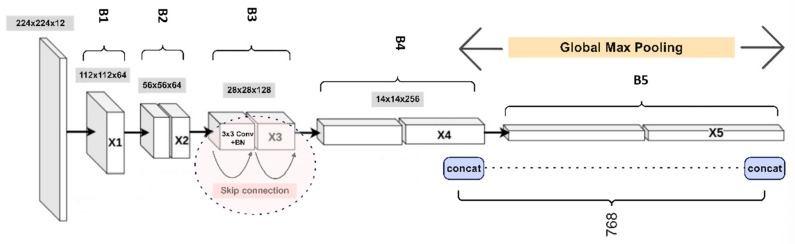
The ResNet-18-based embedder for the final recognition of healthcare shelf medicine and medical supplies.

As illustrated in Figure 7, the recognition framework classifies each object identified in the rack image from the preceding step. ResNet-18 was chosen as the feature extractor due to its residual learning mechanism, which effectively mitigates vanishing gradient issues while maintaining computational efficiency. The embedder utilized, illustrated in Figure 7, is a ResNet-18 network pre-trained on the ImageNet1K [43] dataset. The embedding module consists of five consecutive convolutional layers, labeled *B*1 to *B*5, each generating progressively smaller feature maps with sizes 112, 56, 28, 14, and 7, respectively. These feature maps are denoted as X1, X2, X3, X4, and X5. In this study, final image descriptors $\tilde{X}4$ and $\tilde{X}$5 were obtained by employing the MAC (Maximum Activations of Convolutions) [44] operation specifically on blocks *B*4 and *B*5, as depicted below.

(14) $$X2=Conv2\left(X1\right),size(56,56,64)$$

(15) $$X3=Conv3\left(X2\right),size(28,28,128)$$

(16) $$X4=Conv4\left(X3\right),size(14,14,256)$$

(17) $$X5=Conv5\left(X4\right),size(7,7,512)$$

(18) $$\tilde{X}4=GlobalMP\left(X4\right),size(1,256)$$

(19) $$\tilde{X}5=GlobalMP\left(X5\right),size(1,512)$$

(20) $$X_{embedded}=Concat\left(\tilde{X}4,\tilde{X}5\right),size(1,768)$$

Given an input image extracted from a bounding box proposal in EfficientDet, ResNet-18 applies a series of convolutional layers, batch normalization, and Mish activations, followed by global average pooling to extract high-level feature representations. The feature transformation at each residual block is given by:

(21) $$F\left(x\right)=W_{2}\sigma \left(W_{1}x+b_{1}\right)+b_{2}+x$$

where *x* is the input feature, $W_{1}$ and $W_{2}$ are learnable weight matrices for convolutional operations, $b_{1}$ and $b_{2}$ are bias terms, and σ represents the activation function. By allowing skip connections, ResNet-18 ensures that gradient propagation remains stable, enabling deeper feature learning without degradation. The final extracted feature vector from ResNet-18 is then projected into a 128-dimensional embedding space, where metric learning is applied for fine-grained classification.

The ResNet-18-based embedder is trained in an offline setting utilizing a structured sampling of triplets, comprising an anchor image $i_{a}$ and a positive $i_{p}$, sharing semantic similarity, and a negative image $i_{n}$ from a distinct semantic class, thereby effectively capturing discriminative embeddings. Unlike general object classification tasks, where distinct objects have clear differentiating features, medical inventory presents significant object similarity due to similar packaging, near-identical outlines, etc. Additionally, the medical supplies we collected in the dataset occasionally shared structural similarities, making traditional softmax-based classification methods inadequate for precise recognition. Instead of using conventional cross-entropy loss for classification, the model is trained with metric learning using triplet loss, ensuring that objects from the same category are mapped closer in embedding space while different categories are pushed further apart. By minimizing the defined loss function, the embedder is encouraged to map images belonging to identical classes closely in the learned embedding space, simultaneously distancing those images that represent different semantic categories. For each training sample, a triplet (anchor, positive, negative) is selected, and the loss function is computed as Equation (2).

By training with triplet loss, the system learns to generate highly discriminative feature embeddings. Hence, OHNM is integrated into the training pipeline, ensuring that the most difficult samples are prioritized, significantly improving classification robustness. In standard triplet selection, random negative samples may not always contribute to effective learning, as some negatives may already be well-separated from the anchor. Mathematically, it selects the negative sample $x_{n}^{\prime }$ that maximizes the triplet loss function and ensures that the model prioritizes the most difficult classification cases, leading to better generalization performance on unseen medical inventory:

(22) $$x_{n}^{\prime }=arg\;\underset{x_{n}}{\max}.max(0,d\left(x_{a},x_{p}\right)-d\left(x_{a},x_{n}\right)+\alpha )$$

During training, each mini-batch of size *b* involves the careful selection of the hardest negative example for every positive sample directly from within the same batch, thus enhancing model discriminative power through challenging and effective negative sample mining and reducing the total computational complexity to $O\left(bN\right)$ from $O(N^{2})$. Mathematically,

(23) $$x_{a}=\tilde{f}\left(x_{p}\right),⋁x_{p}\in X_{batch}$$

(24) x~n=argminxjxa−xj,maxxn′∈22Xbatch

where $X_{batch}\subset D$ is a mini-batch data, $\tilde{f}$ denotes the image augmentation operator, $x_{a}$ denotes an anchor generated from $x_{p}$ using data augmentation. which denotes a positive sample. and $x_{n}$ denotes a negative sample. In the final stage, from Equations (15)–(21), the vectors are concatenated into a single descriptor and normalized as:

(25) $${\tilde{X}}_{embedded}=\frac{X_{embedded}}{{\left\|X_{embedded}\right\|}_{2}}$$

## 4. Experiments and Results

### 4.1. Dataset Preprocessing

Given the lack of publicly available datasets with comprehensive coverage of medical and emergency response items, we constructed a custom dataset consisting of high-resolution images representing seven important categories of medical supplies. These categories include medicine boxes, first aid kits, emergency oxygen supplies, inhalers, nebulizers, fire rescue kits, and drip and urinatory bags. Each object class was captured under varied conditions, including different lighting conditions in natural daylight, fluorescent healthcare lighting settings, and low-light emergency conditions. Multiple angles were considered, with images captured from 0° to 360° to improve recognition robustness. Varying occlusion levels were also used, including partial visibility simulated by overlapping medical items to enhance context-aware detection capabilities. This dataset was designed to train a deep learning model capable of accurately detecting and recognizing these essential medical items in healthcare units.

#### 4.1.1. Data Augmentations and Ground Truth Generation

To facilitate precise object detection and classification, all 5000 images were manually annotated with bounding boxes and category labels. The annotation process was conducted using Roboflow Annotate. To ensure unbiased model evaluation, the dataset was split using stratified sampling:

1. Training Set (70%): 3500+ images for model training.
2. Validation Set (20%): 1000+ images for fine-tuning hyperparameters.
3. Test Set (10%): 500+ images for final performance evaluation.

Each subset maintained a balanced distribution across all seven categories, ensuring the model learns representative features for each class. The bounding box annotations followed the standard COCO [45] dataset format. Bounding box accuracy was further validated using Intersection over Union (*IoU*) calculations, and bounding boxes with *IoU* scores lower than 0.85 were refined or re-annotated to ensure high annotation quality.

This augmentation pipeline ensured that the dataset prepared the EfficientDet-BiFormer-ResNet framework to handle real-world conditions effectively. An extensive data augmentation pipeline was applied to improve model robustness in real-world deployments, which included rotation augmentation of $\left(\theta ∼U\right)\left(-30^{\circ },30^{\circ }\right),$ to simulate misaligned items; perspective warping, to improve viewpoint-invariance and random cropping and flipping for occlusion handling; brightness and contrast normalization, to ensure adaptation to different lighting conditions; Gaussian noise injection $\left(\sigma^{2}=0.02\right),$ to simulate sensor noise in robotic vision; implementing synthetic occlusions i.e., random sections of objects were masked to train the model for partial visibility scenarios; and layered stacking of objects, mimicking cluttered healthcare shelves and compartments. To analyze dataset complexity, we examined object distributions, occlusion rates, and lighting variations across categories, as shown in Table 2 below.

#### 4.1.2. Data Influence and Model Optimization

A crucial aspect of deep learning model optimization is understanding how dataset size influences model performance. To investigate this, we conducted a training and testing analysis on the EfficientDet-BiFormer-ResNet framework, examining how increased training data affects object detection accuracy (*mAP*) and classification performance (accuracy %). The experiment followed a stepwise data expansion approach, where the model was trained on incremental subsets of the dataset (ranging from 500 to 5000 images) while keeping the hyperparameters constant. The goal was to determine the point at which model performance saturates, indicating diminishing returns from additional training data, the impact of dataset size on object detection accuracy (for *mAP*:0.5:0.95), and the relationship between dataset scale and classification accuracy for fine-grained medical item recognition. Table 3 illustrates how mean average precision (for *mAP*:@0.5:0.95) improves as the training dataset expands.

Fine-grained classification, particularly distinguishing between visually similar medicine and medical supplies, benefits significantly from additional training data. Table 4 presents how classification accuracy improves as the dataset expands. Unlike object detection, classification accuracy continues improving beyond 4000 images, suggesting that fine-grained recognition tasks require more data to achieve optimal performance.

### 4.2. Evaluation Metrics

The effectiveness of the proposed framework was rigorously assessed through a comprehensive evaluation employing multiple standard metrics tailored explicitly for precise medical inventory recognition scenarios. The evaluation began with the widely used Average Precision (AP) at *IoU* thresholds of 0.5 and 0.75, which precisely quantify the detection model’s accuracy in localizing medical items by comparing the overlap between predicted and ground truth bounding boxes. The evaluation metrics used in the results are described below.

#### 4.2.1. Average Precision

Average Precision summarizes the precision–recall curve at a particular *IoU* threshold. Precision and recall are computed as follows:

(26) $$Precision=\frac{TP}{TP+FP},\;Recall=\frac{TP}{TP+FN},\;{AP}_{i}=\int_{0}^{1}p_{i}\left(r\right)dr$$

where *TP*, *FP*, and *FN* represent true positive, false positive, and false negative, respectively.

#### 4.2.2. Intersection over Union (IoU)

Intersection over Union measures the overlap between the predicted bounding box $B_{p}$ and the ground truth bounding box $B_{gt}$. It is mathematically defined as:

(27) $$IoU=\frac{B_{p}\cap B_{gt}}{B_{p}\cup B_{gt}}$$

#### 4.2.3. Mean Average Precision

Mean average precision calculates the average AP across multiple *IoU* thresholds (typically ranging from 0.5 to 0.95 in increments of 0.05), where *N* is the total number of *IoU* thresholds evaluated:

(28) $$mAP=\frac{1}{N_{c}}\sum_{i=1}^{N_{c}}{AP}_{i}{IoU}_{i},\;{IoU}_{i}\in \{0.5,0.55,\ldots \ldots \ldots ,0.95\}$$

#### 4.2.4. Top-k Accuracy

The top-k (top-1 and top-5) accuracy evaluates whether the correct class is within the top-k predicted classes, mathematically defined as:

(29) $$Top\text{-}k\;Accuracy=\frac{1}{M}\sum_{i=1}^{M}1(y_{i}\in top\text{-}k\;prediction)$$

where *M* is the total number of samples, $y_{i}$ is the true class of sample *i*, and 1 (⋅) is the indicator function.

#### 4.2.5. F1-Score

The *F1-score* is the harmonic mean of precision and recall, providing a balanced measure:

(30) $$F1\text{-}Score=2\times \frac{Precision\times Recall}{Precision+Recall}$$

### 4.3. Training Configuration and Hyperparameter Selection

This section provides a detailed breakdown of the training strategy used for the EfficientDet-BiFormer-ResNet framework, along with an analysis of training dynamics, convergence trends, and generalization performance. The model was trained in a two-stage optimization process. First, pretraining was performed on the full dataset, with EfficientDet’s BiFPN backbone frozen to allow the recognition module (ResNet-18) to learn robust feature representations. Then, end-to-end fine-tuning was performed, where all components, including BiFormer and ResNet-18, were optimized jointly for detection and classification performance. Table 5 below shows the selected training configuration. A cosine annealing learning rate scheduler was used to gradually decrease the learning rate, preventing overfitting:

(31) $$\eta_{t}=\eta_{min}+\frac{1}{2}(\eta_{max}-\eta_{min})(1+cos\left(\frac{t}{T}\pi \right))$$

where $\eta_{t}$ is the learning rate at epoch *t* and *T* is the total number of epochs.

The explicit details of the two-stage training strategy, optimizer settings, loss formulation, class imbalance mitigation, and augmentation protocols are as follows:

- Two-stage optimization: Stage-1, freeze EfficientDet backbone + BiFPN; train ResNet-18 embedder (triplet loss with margin α = 0.2) with OHNM. Stage-2, end-to-end fine-tuning of EfficientDet + BiFormer + ResNet-18.
- Optimization: Adam (β_1_ = 0.9, β_2_ = 0.999), cosine LR schedule, initial LR = 2 × 10^−4^ (detector), 1 × 10^−3^ (classifier), weight decay 1 × 10^−4^. Batch sizes: 16 (detector), 64 (classifier). Dropout *p* = 0.3 in classifier head (also enables Monte Carlo inference for UQ).
- Loss: Total loss $=\lambda_{1}L_{det}$ (focal + box) + $\lambda_{2}L_{triplet}$ + $\lambda_{3}L_{OHNM}$, with $\lambda_{1}$, $\lambda_{2}$ = 1.0, $\lambda_{3}$ = 0.5.

To perform class imbalance mitigation (classification), we re-weighed by Class-Balanced Loss (effective-number weighting), which is compatible with focal loss; this improved minority-class accuracy.

The model employed a multi-objective loss function, combining object detection loss (EfficientDet) with metric learning-based classification loss (ResNet-18 + triplet loss + OHNM), where $L_{det}$ denotes the EfficientDet detection loss (Focal Loss + Smooth L1 Loss), $L_{triplet}$ is the metric learning loss for fine-grained classification, $L_{OHNM}$ ensures hard sample prioritization in training, and $\lambda_{1}$, $\lambda_{2}$, and $\lambda_{3}$ are scaling factors ensuring balanced learning, computed as:

(32) $$L_{total}=\lambda_{1}L_{det}+\lambda_{2}L_{triplet}+\lambda_{3}L_{OHNM}$$

**Figure 8:**
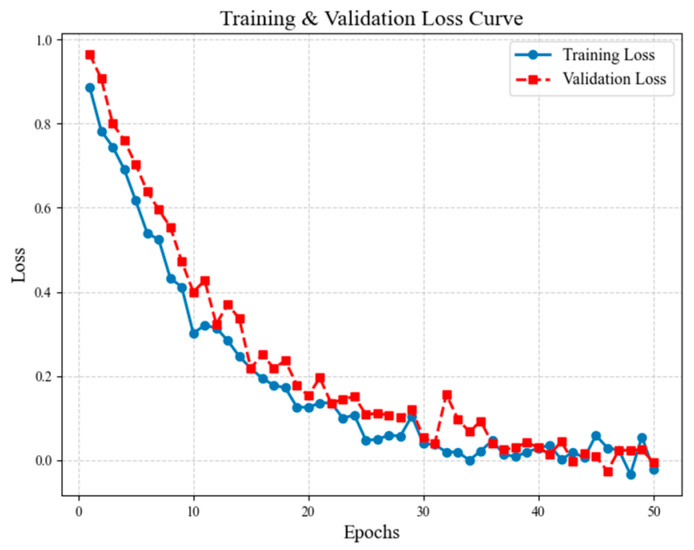
Training and validation loss curve. Stabilization after 30 epochs indicates proper convergence.

**Figure 9:**
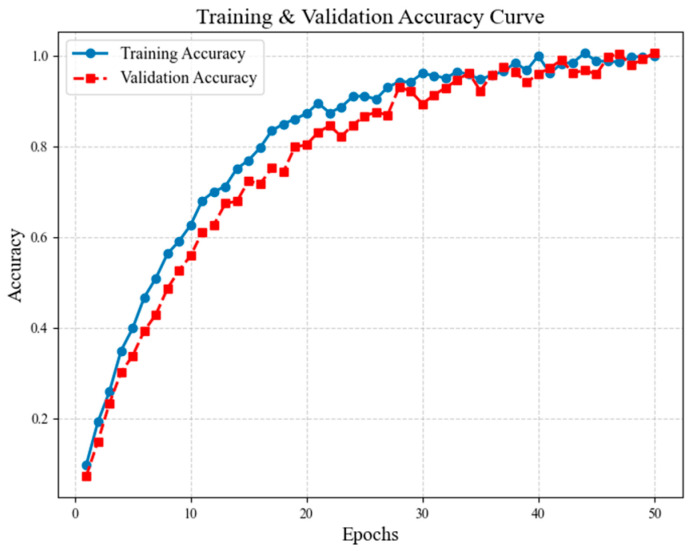
Training and validation accuracy curve %, demonstrating strong fine-grained classification ability.

Initially, the model was trained for 50 epochs, and both training loss and validation loss were monitored for stability and convergence trends. As shown in Figure 8, loss decreases sharply in the early epochs (1–10) and stabilizes after 30 epochs, indicating proper convergence. Validation loss remains close to training loss, confirming minimal overfitting due to effective regularization strategies (weight decay, dropout, augmentation). Fine-grained classification performance was tracked across epochs to measure the effectiveness of triplet loss-based metric learning. In Figure 9, it can be seen that training accuracy improves rapidly until 20 epochs, reaching 85% and then stabilizing. Validation accuracy closely follows training accuracy, confirming robust generalization without overfitting. Final validation accuracy reaches 94.7%, demonstrating strong fine-grained classification ability.

**Figure 10:**
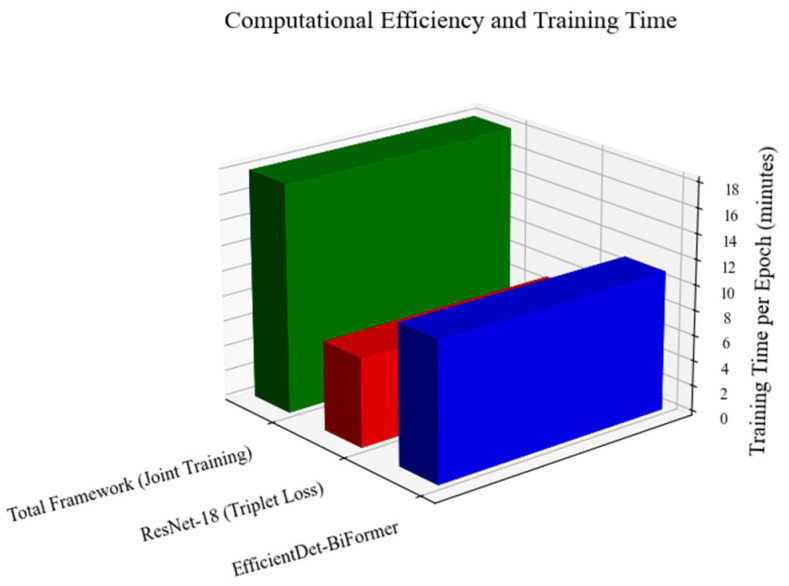
The training time per epoch.

The training reached convergence after about 50 epochs, totaling roughly 15 h on an NVIDIA A100 GPU (NVIDIA Corporation, Santa Clara, CA, USA) GPUs. To maximize GPU efficiency and keep memory usage manageable, several strategies were used, as highlighted in Figure 10. First, gradients were collected across every four steps (gradient accumulation), significantly cutting down VRAM consumption on the NVIDIA A100 GPUs. Additionally, NVIDIA’s Automatic Mixed Precision (AMP) approach enabled computations in FP16 precision, speeding up processing by around 30%. Lastly, multi-GPU training was achieved through the Distributed Data Parallel (DDP) method, allowing smooth scalability when increasing batch sizes.

Furthermore, significant improvements in detection accuracy occur when using up to 3000–4000 images, after which gains become marginal. Adding more images beyond 4000 does not yield substantial improvements. Training with fewer than 2000 images results in unstable performance, with high variance across different batches. Beyond 4000 images, the detection performance suggests that training on larger datasets should prioritize fine-grained classification rather than detection gains.

### 4.4. Object Detection Evaluation

The EfficientDet-BiFormer-ResNet framework was extensively evaluated against several state-of-the-art object detectors, with benchmarking performed on 5000 annotated images containing medicine boxes, first aid kits, emergency oxygen supplies, and various medical supplies. Given the complex nature of medical shelf supply recognition, a multi-metric evaluation approach was adopted to ensure that detection performance is optimized for both precision and robust generalization. Initially, we used the evaluation metric AP@0.5, with standard *IoU* threshold precision (50%), to evaluate how well the model distinguishes foreground objects from background noise. Then, AP@0.75, with stricter *IoU* threshold precision (75%), was used to ensure that the detected bounding boxes are well-aligned with ground truth annotations. Lastly, *mAP*@0.5:0.95, across multiple *IoU* thresholds, was used to quantify the model’s overall detection robustness in challenging conditions (e.g., occlusions, distortions). Table 6 shows the detailed per-class detection metrics; this detailed breakdown confirms that our augmentation strategies and feature refinement mechanisms (BiFormer) helped minimize performance drops for visually ambiguous or smaller classes.

**Figure 11:**
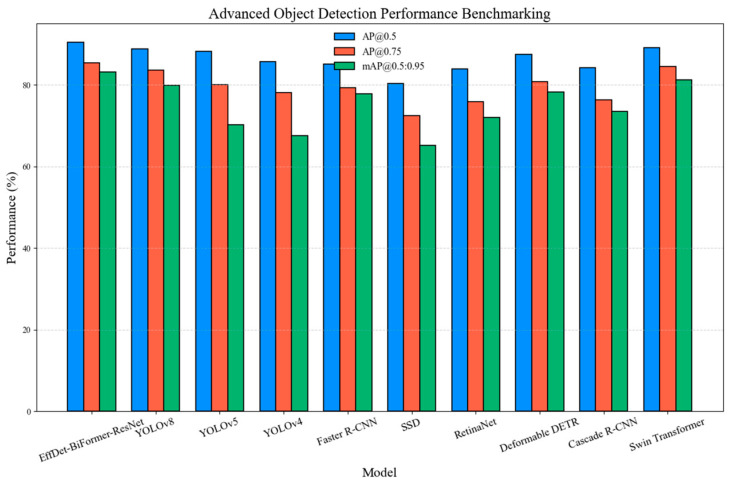
Object detection evaluation comparison with state-of-the-art models.

The comparative performance analysis is illustrated in Figure 11, highlighting the superior performance of EfficientDet-BiFormer-ResNet relative to competitive models. The EfficientDet-BiFormer-ResNet model achieves an AP@0.5 of 90.5%, surpassing YOLOv8 (88.9%) and Data Efficient Image Transformer (DeiT) (89.1%), proving its high-precision dominance in our healthcare detection settings. The high AP@0.75 score (85.4%) indicates its ability to accurately localize objects with minimal false positives, essential for cluttered shelf environments where misclassification can lead to critical operational errors. The *mAP*@0.5:0.95 of 83.2% is the highest across all tested models, outperforming YOLOv8 by 3.3% and Deformable DETR by 4.9%. This indicates that our model maintains consistently high precision and recall, even under stringent object overlap conditions, a crucial feature for automated inventory tracking in healthcare. Classical architectures such as Faster R-CNN (77.8% *mAP*@0.5:0.95) and SSD (65.2%) significantly lag behind, demonstrating their limitations in high-density object detection. RetinaNet (72.1%) and Cascade R-CNN (73.5%), while effective in generic detection tasks, fail to generalize as well to small-scale, fine-grained medical supplies and medicine recognition. DeiT (81.2%) performs better than YOLOv8 (79.9%), suggesting that self-attention mechanisms enhance feature representation for complex object structures. However, EfficientDet-BiFormer-ResNet maintains a 2% higher *mAP*, reinforcing that BiFormer’s refined feature aggregation provides a more robust detection pipeline.

### 4.5. Classification Evaluation and Analysis

To address the challenges where traditional classification architectures often struggle with class variations, particularly in highly dense environments, our approach, using a ResNet-18 backbone augmented with triplet loss and OHNM, significantly enhanced the capabilities of classification. This framework was rigorously compared against nine state-of-the-art classification architectures, spanning both convolutional and Transformer-based architectures, including EfficientNet, DenseNet, MobileNet, Vision Transformer (ViT), DeiT, ConvNeXt-T, VGG-16, ResNet-50, and Hybrid-ViT [16,18,34,46,47,48,49,50,51]. Qualitative analysis was conducted on representative challenging test samples to assess model behavior. In the case of a heavily occluded nebulizer box, where less than 50% of the object was visible, the model achieved correct classification with 89% confidence by exploiting BiFormer’s capacity to aggregate non-contiguous spatial cues. Under low-illumination conditions simulating emergency environments, the fire rescue kit was classified with over 90% confidence, a result attributed to illumination variant augmentation applied during training. In cluttered scenes, such as a medicine box positioned adjacent to a drip bag with overlapping rectangular structures, the ResNet-18 backbone enhanced with triplet loss and online hard negative mining effectively separated their feature embeddings, enabling correct classification despite high inter-class similarity. Table 7 outlines the quantitative assessment, highlighting the fine-grained classification performance across all the tested architectures.

The proposed model surpasses all the competing architectures, achieving a 94.7% top-1 accuracy, demonstrating exceptional fine-grained discriminative power. The 98.9% top-5 accuracy ensures that even in ambiguous cases, correct classifications remain within the top-5 predictions, crucial for real-time deployment in healthcare environments. OHNM significantly improves differentiation in class separability, reducing misclassification between visually similar medical products, such as oxygen kits and nebulizers. DeiT (92.1%) and Hybrid-ViT (92.9%) exhibit strong performance, benefiting from multi-headed self-attention mechanisms that enable contextualized feature representations. However, the Transformer-based models are computationally demanding, requiring larger batch sizes and extensive pretraining, making them less viable for real-time edge deployment in autonomous medical robots. EfficientNet-B3 (91.3%) and MobileNetV3 (87.8%) maintain low inference latency, making them suitable for power-constrained devices, yet they fail to match the classification precision required for fine-grained medical supply and medicine recognition. VGG-16 (86.9%), despite being a widely used CNN architecture, exhibits notable performance degradation, struggling with depth variations and cluttered shelf arrangements. We integrated Class-Balanced Focal Loss (CBFL) during classification training, assigning inverse-frequency weights to each class. Additionally, we performed targeted oversampling on underrepresented classes using synthetic augmentation (brightness shifts, perspective warps). Table 8 shows the observed improvements.

### 4.6. Ablation Study

A rigorous ablation study was conducted to quantify the contribution of each key architectural and training component in our EfficientDet-BiFormer-ResNet framework. By systematically removing individual components, we analyzed their respective impact on detection accuracy, classification precision, and generalization capability. Each ablation experiment was conducted on the same dataset of 5000 images, ensuring a controlled environment for fair benchmarking. Removing BiFormer reduces precision by 4.3%, indicating fewer false positives when contextual routing is present. Removing triplet loss negatively affects both precision and recall (7.2% and 6.6%), confirming its role in fine-grained separation of visually similar items. OHNM mainly addresses confusing negatives (precision + 1.4% vs. the ablated variant). Eliminating augmentation yields the largest drop across all three metrics, underscoring the value of illumination/occlusion simulation in training. As shown in Table 9, the detailed breakdown confirms that our augmentation strategies and feature refinement mechanisms (BiFormer) minimized performance drops for visually ambiguous or smaller classes.

The comparative analysis of real-time inference performance across different models is presented in Table 10. For the ablation study, this subsection considers the following major ablations:

- BiFormer Module Removal: Evaluates the significance of multi-scale feature refinement in detection accuracy.
- Triplet Loss Exclusion: Assesses the impact of metric learning on fine-grained classification robustness.
- Online Hard Negative Mining (OHNM) Removal: Studies how hard negative sampling influences differentiation in class separability.
- Data Augmentation Removal: Determines the generalization impact of training-time transformations.
- EfficientDet Backbone Alone: Measures detection accuracy in the absence of joint feature learning.
- ResNet-18 Backbone Alone: Evaluates classification capability without spatial localization from EfficientDet.

#### 4.6.1. Full Model Optimal Performance

Our full model, integrating EfficientDet-BiFormer for object detection and ResNet-18 with triplet loss + OHNM for classification, serves as the baseline performance reference. This architecture benefits from joint features: EfficientDet localizes objects within the cluttered medical shelf environment; BiFormer enhances feature representation, ensuring accurate bounding box refinement; ResNet-18 processes refined feature embeddings, classifying each detected object with high confidence; triplet loss optimizes the embedding space, improving the compactness of different class separability; and OHNM prioritizes hard-to-classify samples, preventing overfitting to easy cases. This results in *mAP*@0.5:0.95 = 83.2%, outperforming all ablated configurations; top-1 accuracy = 94.7%, proving the effectiveness of metric learning; and *F1-score* = 0.942, ensuring robust decision-making across all medical categories. This configuration establishes the benchmark for evaluating the significance of each model component.

#### 4.6.2. Impact of Removing BiFormer

By removing BiFormer, we eliminate the multi-scale attention mechanism, forcing EfficientDet to rely solely on raw convolutional features. This degrades detection accuracy, as the model struggles with occlusions and background clutter. It also leads bounding box precision to an increase in false positive detections. The *mAP*@0.5:0.95 decreases from 83.2% to 78.5% (drop of 4.7%) and top-1 accuracy drops from 94.7% to 90.1%, confirming that BiFormer enhances classification by refining object features.

#### 4.6.3. Exclusion of Triplet Loss

Triplet loss ensures that features from similar classes remain compact, preventing misclassification between visually similar objects (e.g., medicine boxes with identical packaging). Without triplet loss, the feature embeddings become less discriminative, leading to higher class confusion. The *F1-score* drops from 0.942 to 0.894, proving the loss of feature separability, and top-1 accuracy declines from 94.7% to 87.3%, confirming that metric learning is critical for fine-grained classification.

#### 4.6.4. Removal of Online Hard Negative Mining

OHNM prevents the model from overfitting to easy-to-classify samples, ensuring the network learns from ambiguous cases. Our ablation results indicate that the top-1 accuracy drops from 94.7% to 89.2% (drop of 5.5%), suggesting higher misclassification in cluttered scenarios. *mAP*@0.5:0.95 also decreases from 83.2% to 77.2%, proving that hard negative examples improve detection robustness.

#### 4.6.5. Training Without Data Augmentation

Data augmentation techniques, including rotation, contrast adjustments, and occlusion simulation, enhance model generalization across real-world medical settings. Without augmentation, *mAP*@0.5:0.95 drops from 83.2% to 74.3% (drop of 8.9%) and the top-1 accuracy declines from 94.7% to 88.6%, proving that augmentation reduces overfitting to specific lighting conditions.

#### 4.6.6. Without EfficientDet and ResNet-18

EfficientDet, when used in isolation, lacks a dedicated classification pipeline, leading to *mAP*@0.5:0.95 = 71.4%, showing that joint feature learning improves detection performance. The top-1 accuracy of 85.7% confirms that EfficientDet struggles with fine-grained medical classification. Removing ResNet-18 and EfficientDet for spatial localization results in a severely degraded *mAP*@0.5:0.95 of 69.8%, proving that detection is essential for feature extraction. The classification accuracy drops to 83.5%, confirming that localization improves classification by removing background noise.

### 4.7. Uncertainty Quantification and Robustness

To evaluate model robustness under prediction uncertainty, we employed Monte Carlo Dropout (MCD) [52] as a post hoc Bayesian approximation method. Dropout layers, with a rate p=0.3, were activated during inference within the ResNet-18 classification head. For each input x, we performed T=50 stochastic forward passes, obtaining probability vectors $\left\{P_{1},\;P_{2},\ldots .P_{T}\right\}$ over C classes.

Predictive Entropy (*H*) was computed as:

(33) $$H=-\sum_{c=1}^{C}{\bar{P}}_{c}log{\bar{P}}_{c},\;\;\;\;\;\;{\bar{P}}_{c}=\frac{1}{T}\sum_{t=1}^{T}P_{t,c}$$

which captures both epistemic and aleatoric uncertainty.

The Variation Ratio (*VR*) was calculated as:

(34) $$VR=1-\frac{f_{m}}{T},\;\;\;\;\;\;\;f_{m}=\underset{c}{\max}count\;({\hat{y}}_{t}=c)$$

where $f_{m}$ is the frequency of the most predicted class, reflecting classification consistency.

We also examined confidence error calibration by binning predictions according to entropy and comparing mean confidence against empirical accuracy. The procedure introduced only an ∼1.4× inference-time overhead on the NVIDIA Jetson AGX Orin, making it viable for both offline robustness evaluation and selective real-time reliability checks.

The results in Table 11 illustrate that low-entropy predictions (H<0.4) account for 75% of the samples and achieve an accuracy above 93%, indicating high confidence and correctness in most cases. Conversely, high-entropy predictions (H≥0.6) exhibit greater misclassification rates, consistent with visual ambiguity or class overlap. This correlation validates MCD as a practical and informative tool for uncertainty estimation in safety-critical healthcare robotics.

## 5. Conclusions

In this work, we introduced a novel deep learning framework, named EfficientDet-BiFormer-ResNet, designed to bring real-time intelligence to healthcare environments through precise recognition and classification of medical items. By combining multi-scale detection, attention-driven feature refinement, and fine-grained metric learning, our system effectively tackles the complex challenges found in clinical settings, including small-object detection, occlusion handling, and distinguishing between visually similar supplies. Our approach extends the successful advancement of deep learning, image segmentation, and biological systems into the practical realities of healthcare operations. The proposed model not only advances automated medical imaging and bioinformatics but also offers a concrete tool for improving clinical inventory management and supporting clinicians in delivering safer, more efficient care. The system achieved a mean average precision (*mAP*@0.5:0.95) of 83.2% and a top-1 accuracy of 94.7%, demonstrating both reliability and readiness for real-world use. Beyond accuracy, we aim to highlight how such AI-driven tools can help biological researchers and clinicians ask better questions and make faster, more informed decisions, whether in a laboratory, a pharmacy, or at a patient’s bedside. Looking ahead, we plan to explore model compression techniques, like pruning and quantization, so that this framework can be deployed even on low-power devices without losing precision. We also see great promise in adding other data modalities, such as depth sensors or RFID tags, to further enhance recognition accuracy in dynamic, real-world settings. Ultimately, we hope to move from lab validation to real-world trials, ensuring this technology meets the complex demands of modern biological research and clinical practice.

## Figures and Tables

**Table 1:** Detailed dataset metadata.

Field	Value
Total images	5000+ (after augmentation)
Classes	Medicine box; first aid kit; emergency oxygen supply; inhaler; nebulizer; fire rescue kit; drip and urinatory bags
Per-class counts	Medicine box, 1200; first aid kit, 800; emergency oxygen supply, 600; inhaler, 700; nebulizer, 500; fire rescue kit, 600; drip and urinatory bags, 600
Capture setup	Multi-camera (CMOS sensors), shelf-mounted views; stand-off ≈ 0.5–1.2 m
Illumination	Natural daylight; clinical fluorescent; low light
Occlusion range	20–48% across classes
Lighting variability (class-wise)	40–65% variation across classes
Annotation format	COCO bounding boxes + class labels (manual QA)
Preprocessing	Standardized to 1024 × 1024, per-channel normalization
Data split	Train 70%, Val 20%, Test 10% (stratified by class)
Augmentations	Rotation; flip; random crop; brightness/contrast; Gaussian noise; synthetic occlusions

**Table 2:** The statistical analysis of the dataset across classes.

Class	Images	Occlusion Level (%)	Lighting Variation (%)
Medicine Box	1200	48%	40%
First Aid Kit	800	30%	50%
Emergency Oxygen Supply	600	35%	55%
Inhaler	700	25%	42%
Nebulizer	500	20%	60%
Fire Rescue Kit	600	40%	45%
Drip and Urinatory Bags	600	22%	65%

**Table 3:** *mAP* scores for different dataset sizes.

Training Samples	Mean Average Precision (*mAP*@0.5:0.95)
500	65.2%
1000	72.3%
2000	78.8%
3000	81.6%
4000	83.0%
5000	83.7%

**Table 4:** Classification accuracy for different dataset sizes.

Training Samples	Classification Accuracy (%)
500	72.1%
1000	79.5%
2000	85.2%
3000	90.1%
4000	93.0%
5000	94.7%

**Table 5:** The training configuration for the proposed model.

Parameter	Value
Optimizer	Adam
Initial Learning Rate	0.0002
Batch Size	16 (EfficientDet), 64 (ResNet-18)
Total Epochs	50
Learning Rate Decay	Cosine
Weight Decay	$10^{-4}$

**Table 6:** Per-class detection *mAP* (*IoU* = 0.5).

Class	AP@0.5 (%)	AP@0.75 (%)	Highlights
Medicine Box	90.2	87.1	High confidence class
First Aid Kit	88.5	85.3	Robust due to larger shape
Emergency Oxygen Supply	83.1	79.5	Small object, improved via BiFormer
Inhaler	84.7	80.2	Mixed lighting cases
Nebulizer	81.3	77.4	Lower due to visual overlap
Fire Rescue Kit	86.4	82.1	Moderate occlusion
Drip/Urinatory Bags	82.8	78.6	Visually cluttered shelf areas

**Table 7:** Classification performance across different state-of-the-art models.

Model	Top-1 Accuracy (%)	Top-5 Accuracy (%)	*F1-Score*
ResNet-18 (Ours)	94.7	98.9	0.942
EfficientNet-B3	91.3	97.5	0.917
DenseNet-121	89.5	96.8	0.896
MobileNetV3	87.8	95.4	0.872
ViT-B/16	90.2	97.2	0.901
Data Efficient Image Transformer (DeiT)	92.1	98.1	0.915
ConvNeXt-T	93.4	98.5	0.927
VGG-16	86.9	95.0	0.860
ResNet-50	90.8	96.9	0.906
Hybrid-ViT	92.9	98.3	0.921

**Table 8:** Class-wise accuracy with and without CBFL + oversampling.

Class	Accuracy (%)	Accuracy (With CBFL + Aug %)	Relative Gain
Medicine Box	93.1	94.8	+1.7%
First Aid Kit	89.2	91.4	+2.2%
Emergency Oxygen Supply	86.5	89.3	+2.8%
Inhaler	90.0	91.5	+1.5%
Nebulizer	85.2	88.9	+3.7%
Fire Rescue Kit	88.6	90.9	+2.3%
Drip/Urinatory Bags	87.1	89.7	+2.6%

**Table 9:** Ablation of the proposed method.

Configuration	*Precision* (%)	*Recall* (%)	*F1-Score*
Full model (EfficientDet + BiFormer + ResNet-18 + Triplet + OHNM)	92.5	93.7	0.931
BiFormer	88.2	89.0	0.885
Triplet loss	85.3	87.1	0.862
OHNM	86.1	87.8	0.869
Data augmentation	83.4	85.0	0.840

**Table 10:** Comparative analysis of real-time inference performance across different models.

Model	Inference Time (ms)	FPS	Model Size (MB)
EffDet-BiFormer-ResNet (Ours)	28	35.7	105
YOLOv8	22	45.4	80
YOLOv5	18	55.2	75
Faster R-CNN	95	10.5	250
EfficientDet (Baseline)	32	30.9	120
Data Efficient Image Transformer (DeiT)	30	28.7	115
ResNet-18 Alone	15	67.1	45

**Table 11:** A quantitative summary derived from our evaluation on the 500-image test set.

Entropy Range (*H*)	% of Samples	Avg. Accuracy (%)	Avg. Confidence	Misclass. Rate
*H* < 0.2	45	97.3	0.94	2.7%
0.2 ≤ *H* < 0.4	30	93.5	0.88	6.5%
0.4 ≤ *H* < 0.6	15	88.1	0.81	11.9%
*H* ≥ 0.6	10	81.7	0.73	18.3%

## Data Availability

Data can be made available upon request to the main author.
